# Global Analysis of Biomineralization Genes in *Magnetospirillum magneticum* AMB-1

**DOI:** 10.1128/msystems.01037-21

**Published:** 2022-01-25

**Authors:** Hayley C. McCausland, Kelly M. Wetmore, Adam P. Arkin, Arash Komeili

**Affiliations:** a Department of Molecular & Cell Biology, University of California, Berkeley, California, USA; b Environmental Genomics and Systems Biology Division, Lawrence Berkeley National Laboratory, Berkeley, California, USA; c Department of Plant & Microbial Biology, University of California, Berkeley, California, USA; United States Naval Research Laboratory

**Keywords:** magnetotactic bacteria, biomineralization, RB-TnSeq

## Abstract

Magnetotactic bacteria (MTB) are a phylogenetically diverse group of bacteria remarkable for their ability to biomineralize magnetite (Fe_3_O_4_) or greigite (Fe_3_S_4_) in organelles called magnetosomes. The majority of genes required for magnetosome formation are encoded by a magnetosome gene island (MAI). Most previous genetic studies of MTB have focused on the MAI, using screens to identify key MAI genes or targeted genetics to isolate specific genes and their function in one specific growth condition. This is the first study that has taken an unbiased approach to look at many different growth conditions to reveal key genes both inside and outside the MAI. Here, we conducted random barcoded transposon mutagenesis (RB-TnSeq) in *Magnetospirillum magneticum* AMB-1. We generated a library of 184,710 unique strains in a wild-type background, generating ∼34 mutant strains for each gene. RB-TnSeq also allowed us to determine the essential gene set of AMB-1 under standard laboratory growth conditions. To pinpoint novel genes that are important for magnetosome formation, we subjected the library to magnetic selection screens under varied growth conditions. We compared biomineralization under standard growth conditions to biomineralization under high-iron and anaerobic conditions, respectively. Strains with transposon insertions in the MAI gene *mamT* had an exacerbated biomineralization defect under both high-iron and anaerobic conditions compared to standard conditions, adding to our knowledge of the role of MamT in magnetosome formation. Mutants in an ex-MAI gene, *amb4151*, are more magnetic than wild-type cells under anaerobic conditions. All three of these phenotypes were validated by creating a markerless deletion strain of the gene and evaluating with TEM imaging. Overall, our results indicate that growth conditions affect which genes are required for biomineralization and that some MAI genes may have more nuanced functions than was previously understood.

**IMPORTANCE** Magnetotactic bacteria (MTB) are a group of bacteria that can form nano-sized crystals of magnetic minerals. MTB are likely an important part of their ecosystems, because they can account for up to a third of the microbial biomass in an aquatic habitat and consume large amounts of iron, potentially impacting the iron cycle. The ecology of MTB is relatively understudied; however, the cell biology and genetics of MTB have been studied for decades. Here, we leverage genetic studies of MTB to inform environmental studies. We expand the genetic toolset for studying MTB in the lab and identify novel genes, or functions of genes, that have an impact on biomineralization.

## INTRODUCTION

Magnetotactic bacteria (MTB) are a diverse group of bacteria capable of producing intracellular organelles called magnetosomes (1–7) after taking up iron from the surrounding environment. Magnetosomes are membrane-bound compartments in which biomineralization of magnetic crystals of magnetite (Fe_3_O_4_) and/or greigite (Fe_3_S_4_) occurs (4). Crystals are organized into linear chains along the long axis of the cell, forming a magnetic dipole that allows the cell to orient to Earth’s magnetic fields (8). MTB inhabit low-oxygen environments and are typically found at the oxic-anoxic transition zone (OATZ) in a water column (4). Navigation along magnetic field lines is thought to allow cells to efficiently locate the OATZ, a process called magnetoaerotaxis (9).

MTB are ubiquitous in aquatic environments and can account for up to 30% of microbial biomass in some habitats (5). Because of their presence in water and the large amounts of iron that each cell captures in the process of biomineralization, it is likely that MTB have a large impact on iron cycling in the ocean, potentially taking up anywhere from 1 to 50% of dissolved iron inputs into the ocean (10). However, most of what we know about MTB in the environment comes from surveys of species and their numbers in particular habitats. The dynamic responses of MTB, particularly at a genetic level, to fluctuations in the environment remain largely unexplored. A greater understanding of the molecular mechanisms of biomineralization and iron sequestration by MTB in response to changing conditions will inform environmental studies, including the impact that MTB have on iron cycling. However, most of our knowledge of the genetics and cell biology of MTB comes from studies done under static conditions that do not accurately reflect the natural environments under which MTB grow. Here, we take advantage of the well-studied model organism *Magnetospirillum magneticum* AMB-1 and develop a high-throughput genetic strategy to connect genetics to environmental changes.

The genes needed to produce magnetosomes are in magnetosome gene clusters (MGCs). In some species, like the model organisms AMB-1 and Magnetospirillum gryphiswaldense, the MGCs are discrete magnetosome gene islands (MAIs). The MAIs in AMB-1 and Magnetospirillum gryphiswaldense MSR-1 are well characterized (11, 12). Both organisms have approximately 100 genes in their respective MAIs. However, it was shown by Kolinko et al. that only 31 of the genes in the MSR-1 MAI are necessary and sufficient to synthesize magnetosomes, although even fewer may be required (13). This implies that the other ∼70 genes in the MAIs are unnecessary for the formation of magnetosomes, are redundant with other genes, or are only required under certain growth conditions. There is evidence that some MAI genes are only required for magnetosome formation under certain growth conditions. For example, the genes *mamX* and *ftsZm* are necessary under oxygen-reducing conditions but not nitrate-reducing conditions (14, 15).

Genes outside the MSR-1 MAI (ex-MAI genes) have also been connected to magnetosome formation as growth conditions are changed. Deletion of the *nap* operon in MSR-1 resulted in the formation of small, poorly aligned magnetosomes (16), indicating that nitrate metabolism, while not necessary for the growth of MSR-1, is critical in the formation of magnetosomes. The possibility that other genes, both inside and outside the respective MAIs of MSR-1 and AMB-1, are required for magnetosome formation under specific conditions has not been thoroughly examined (17–21).

Previous genetic studies in magnetotactic bacteria have used either targeted reverse genetics to determine the role of individual genes or operons or forward genetic screens that focus solely on clear magnetosome mutants. Here, we present a global and readily scalable approach to investigate the genetic requirements of AMB-1. We used a screening technique called random barcoded transposon site sequencing (RB-TnSeq) (22), which involves generating a pooled library of tens of thousands of transposon mutants. First, we used the RB-TnSeq library to determine the essential gene set of AMB-1 under our standard growth conditions, providing a broader view of the lifestyle of this important model organism. We then used the RB-TnSeq library to conduct a high-throughput magnetic selection to study the genetic requirements for magnetosome formation under multiple environmental conditions. In particular, we focused on magnetosome formation in various oxygen and iron concentrations, both of which have been shown to influence magnetosome formation (23, 24). Magnetic selection experiments uncovered new functions of known magnetosome genes while also revealing novel ex-MAI genes that may have a role in magnetosome formation.

## RESULTS

### RB-TnSeq libraries reveal the essential gene set of AMB-1.

**FIG 1:**
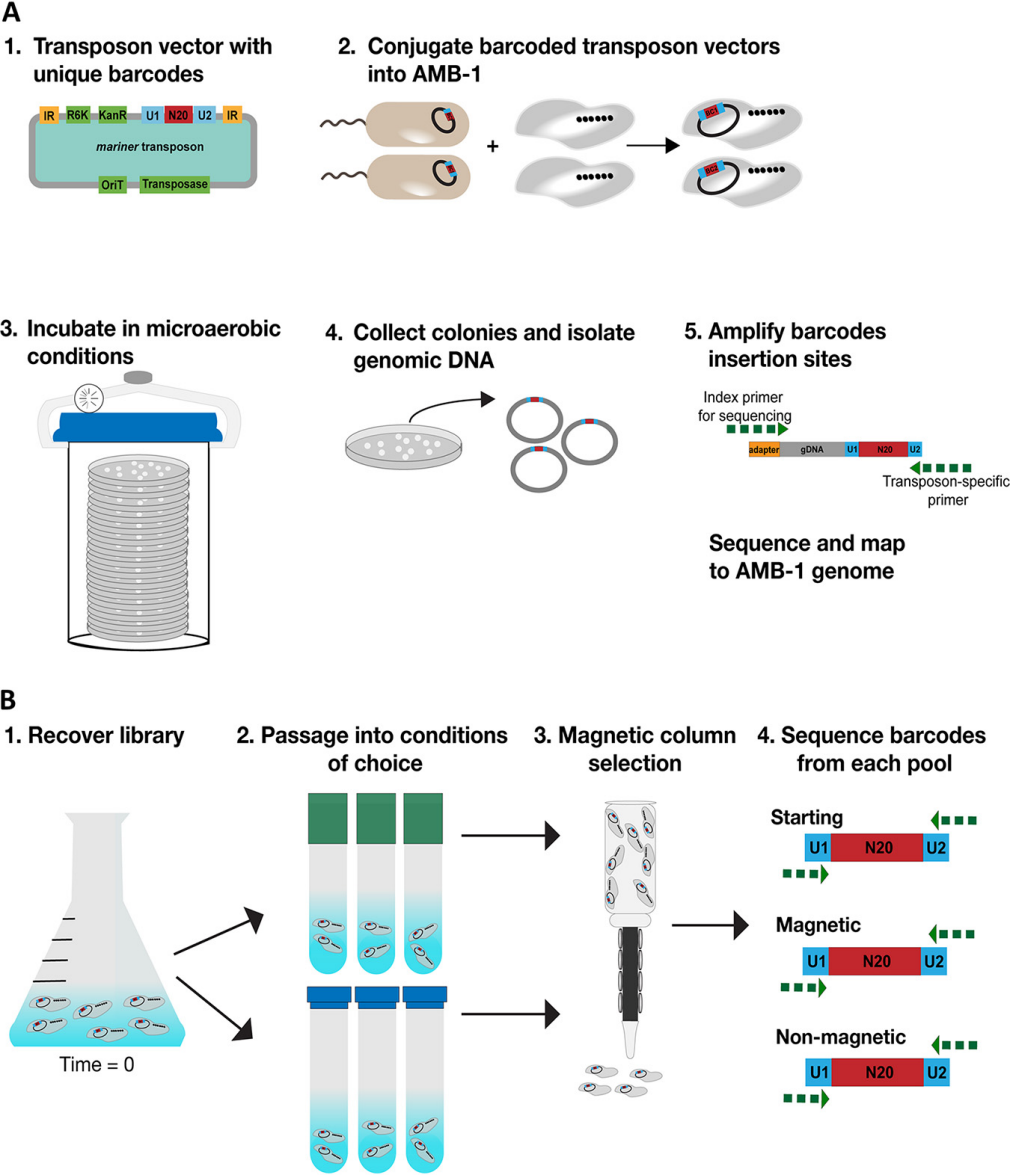
RB-TnSeq and magnetic selection. (A) Flow chart of RB-TnSeq library construction and sequencing. (B) Flow chart of magnetic selection using RB-TnSeq library.

To conduct high-throughput genetic screens in AMB-1, we generated pooled transposon insertion libraries containing thousands of mutants using the transposon mutagenesis technique, RB-TnSeq (22) (Fig. 1). A library of ∼30 million *mariner* transposon vectors (APA752) in the Escherichia coli host strain WM3064 was conjugated with AMB-1. After growth on selective plates, all colonies were pooled for sequencing. The location of each transposon was mapped to the AMB-1 genome using Illumina sequencing.

**FIG 2:**
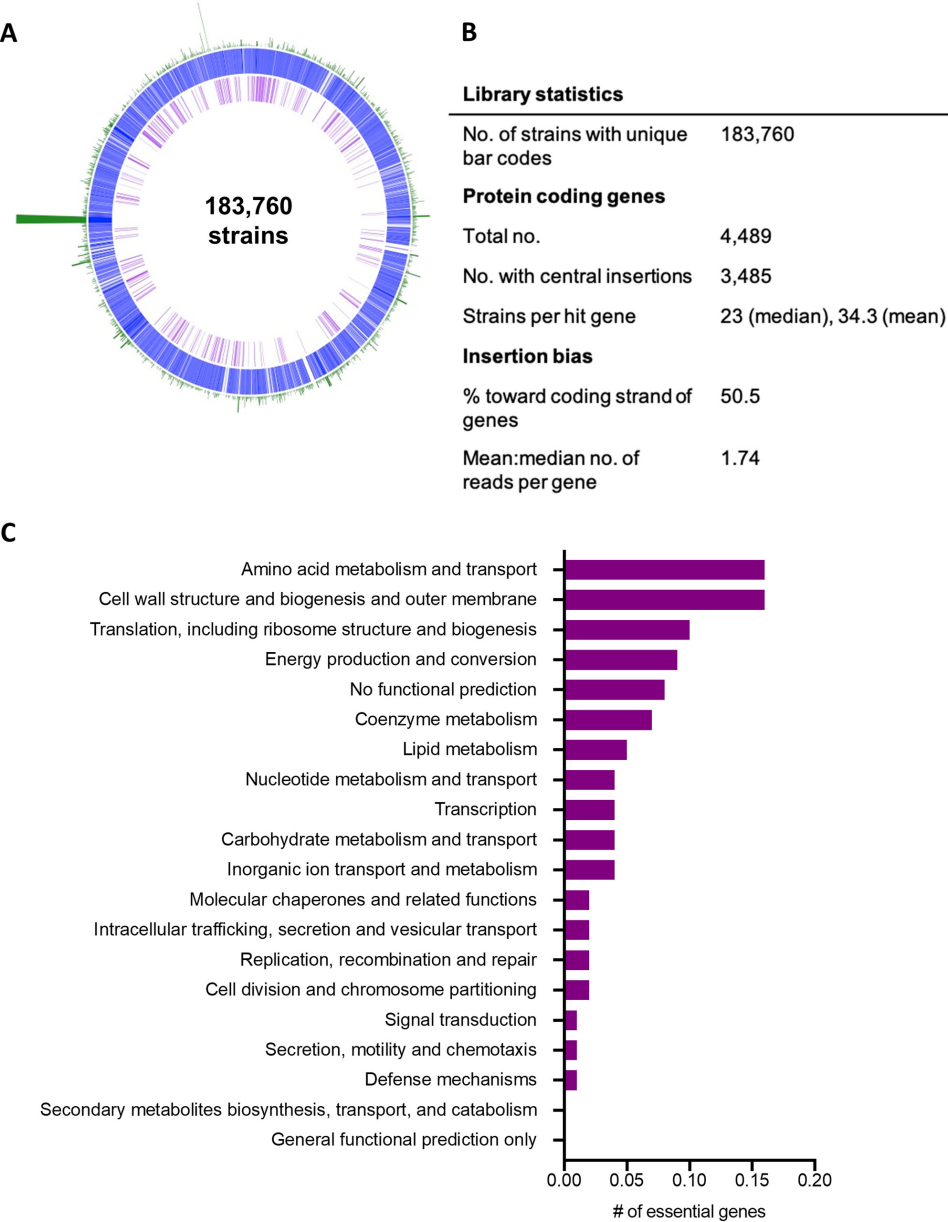
RB-TnSeq libraries and essential gene sets. (A) Map of WT AMB-1 RB-TnSeq library showing AMB-1 genome (blue), WT essential genes (purple), and transposon insertion loci and densities (green). (B) Summary of library statistics for ML2. (C) Genes essential to the ML2 library under standard growth conditions grouped into COG categories.

We generated a successful library in wild-type (WT) AMB-1 called magnetotactic bacteria library ML2 (ML2). The library was constructed under our standard laboratory conditions: MG medium supplemented with 30 μM iron and grown microaerobically. ML2 contained 183,760 unique barcodes (Fig. 2A and B). There were 34.3 hits per protein on average (mean). The read bias (mean-median reads per hit protein) of 1.74 indicates a moderate bias in the library, where no genes are underrepresented by more than 2-fold, on average. Since ML2 has a large number of mutant strains with broad coverage of the genome, it was used for downstream biomineralization screens.

Another benefit of making an RB-TnSeq library is that it can be used to determine the putative essential gene set of a strain under the conditions under which it was made (Fig. 2A). Essential genes have very few or no insertions because those mutants should not be viable. Here, we determined the essential gene set of ML2 under standard laboratory conditions. We identified 445 essential genes in the AMB-1 genome, ∼9.9% of protein coding genes, which is similar to the percentage of essential genes in other RB-TnSeq libraries (25). The remaining 4,216 genes are either nonessential (3,774 genes) or were not included in the list of essential genes (472 genes). Those genes that were not included are very similar to other regions of the genome or less than 800 nucleotides. Genes larger than 800 nucleotides were determined to be unlikely to have no insertions by chance (25).

Binning the essential genes into COG categories showed that amino acid metabolism and cell wall structure/biogenesis accounted for the largest proportion of essential genes (16% each) (Fig. 2C). Translation (10% of essential genes) and energy production (9% of essential genes) also contained a large proportion of essential genes. While these categories are expected for essential genes, the individual genes within each category can provide information about what MTB need to grow. For example, a succinate dehydrogenase subunit (*amb3952* or AMB_RS20000) and tartrate dehydrogenase (*amb3176* or AMB_RS16020) are essential under standard conditions, indicating the role of the tricarboxylic acid cycle in heterotrophic metabolism of AMB-1. Additionally, nitrate reductase subunits (*amb0531* and *amb0533* or AMB_RS02735 and AMB_RS02745), nitrate ABC transporter (*amb0534* or AMB_RS02750), and nitrate-sulfonate-bicarbonate ABC transporter ATP-binding protein (*amb0535* or AMB_RS02755) are essential, where nitrate was the only alternative electron acceptor available for respiration.

### Magnetic selection reveals genes important for biomineralization under high-iron conditions.

**FIG 3:**
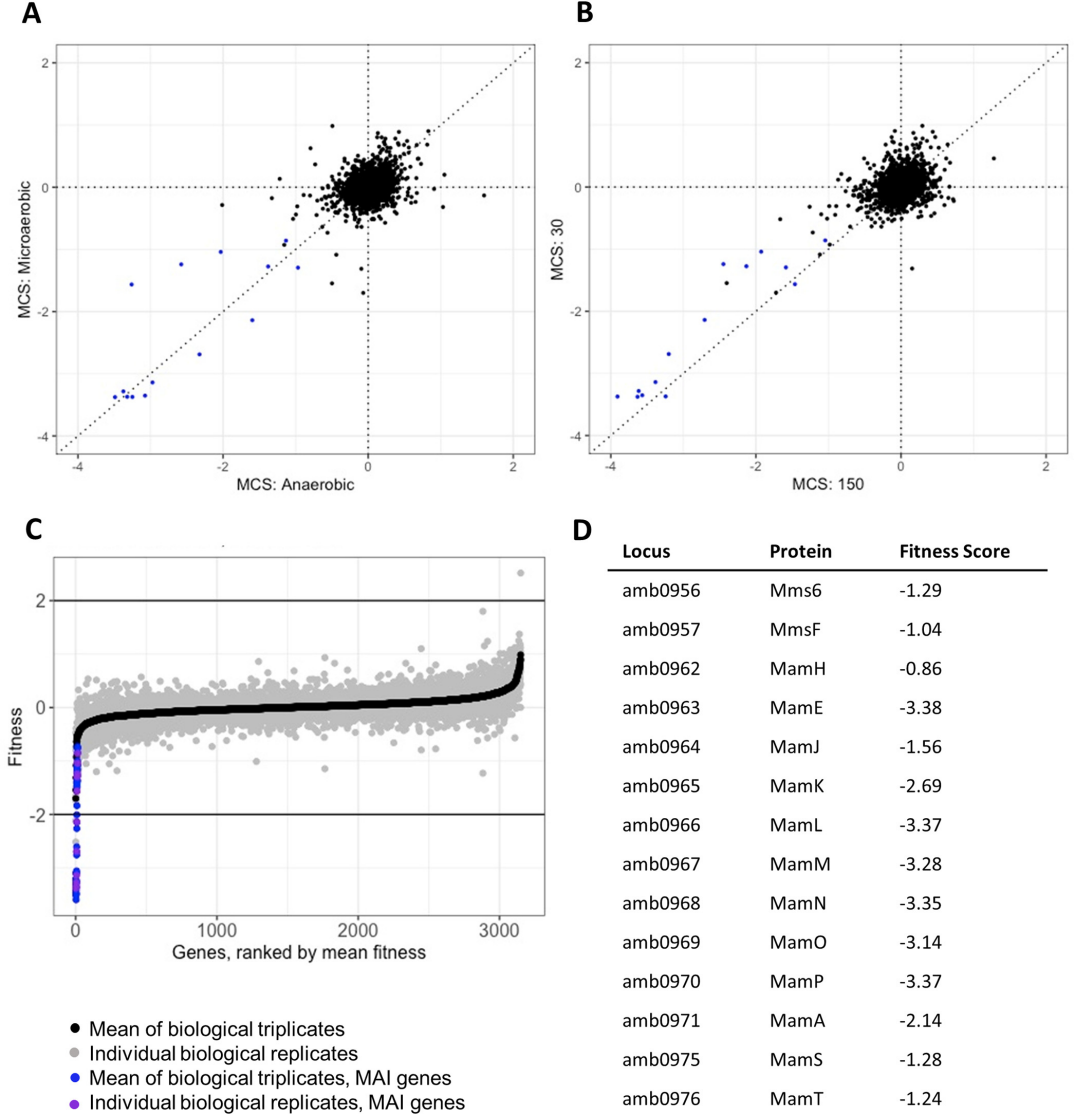
Magnetic fitness of AMB-1 genes. (A) Comparison of magnetic fitness scores for AMB-1 grown under microaerobic (<10% oxygen) or anaerobic (0% oxygen) conditions. Each point is the mean gene fitness of three biological replicates. MAI genes with low MCS highlighted in blue. (B) Comparison of magnetic fitness scores for AMB-1 grown in 30 μM iron or 150 μM iron. Each point is the mean gene fitness of three biological replicates. MAI genes with low MCS are highlighted in blue. (C and D) Magnetosome island gene scores validate the magnetic selection process. (C) Distribution of magnetic fitness scores for the WT grown under standard laboratory conditions (microaerobic [<10% oxygen] and 30 μM iron). MAI genes with low MCS highlighted in blue/purple. (D) Table highlighting mean fitness scores for MGC genes and their respective proteins.

To screen for mutants with defects in biomineralization, we used a magnetic column to separate magnetic from nonmagnetic cells in the RB-TnSeq library (Fig. 1B). After thawing an aliquot of ML2 and growing to late log phase (optical density at 400 nm [OD_400_] of ∼0.150), cells were passaged into growth conditions of choice and allowed to grow to stationary phase (OD_400_ of ∼0.250). Each culture then was filtered through a magnetic column lined with magnets. Both the nonmagnetic and magnetic samples were collected. A sample of the precolumn culture was saved as a control. BarSeq (sequencing of the unique barcodes) was performed on magnetic, nonmagnetic, precolumn, and time zero samples, and then a magnetic column score (MCS) for each gene was calculated based on each strain’s abundance (Fig. 3). It should be noted that BarSeq experiments are usually used to measure growth of each strain based on a strain’s abundance in each experiment. Here, we have adapted the measure of strain fitness to evaluate biomineralization capabilities based on abundance of each strain in precolumn, magnetic, and nonmagnetic samples. To account for any growth defects that might affect MCS, we normalized by subtracting precolumn gene MCS from magnetic and nonmagnetic gene MCS before comparing to time zero. Raw fitness scores for all experiments can be viewed in the Fitness Browser (http://fit.genomics.lbl.gov/cgi-bin/org.cgi?orgId=Magneto) (25).

To validate the screen, ML2 was run over the column after microaerobic growth in 30 μM iron. There were too few cells in the nonmagnetic sample for preparation of genomic DNA (gDNA) because there are very few insertions that completely abolish biomineralization. Accordingly, we reasoned that strains with transposon insertions in magnetosome formation genes would be depleted in the magnetic sample. Thus, a low MCS would correspond to a defect in biomineralization (Fig. 3D). Accordingly, we found that strains with transposon insertions in MAI genes with known roles in magnetosome formation, *mms6* (AMB_RS04895), *mmsF* (AMB_RS04900), *mamH* (AMB_RS04920), *mamE* (AMB_RS04930), *mamJ* (AMB_RS23165), *mamK* (AMB_RS04945), *mamL* (AMB_RS04950), *mamM* (AMB_RS04955), *mamN* (AMB_RS04960), *mamO* (AMB_RS04965), *mamP* (AMB_RS04970), *mamA* (AMB_RS04975), *mamS* (AMB_RS04995), and *mamT* (AMB_RS05000), were underrepresented in the populations of magnetic cells (Fig. 3D). Three genes, *mamQ*, *mamR*, and *mamB*, that are known to have a role in magnetosome formation are replicated exactly in the MAI downstream of the *mamAB* operon (11), and strains with insertions in those genes did not appear in our screen. It is interesting that *mamJ* and *mamK*, which are involved in magnetosome chain alignment, not biomineralization, also had negative MCS under these conditions. Perhaps uneven chain segregations in these mutants yield a small but detectable subpopulation that cannot bind to the magnetic column.

The goal of further screens was to identify genes involved in biomineralization when cells are grown under alternative conditions. We focused on genes required for biomineralization under anaerobic conditions and under high-iron conditions. Recent work by Amor et al. has shown that the concentration of iron supplied in the growth medium affects both iron uptake and magnetite formation in AMB-1 (10). As iron concentration is increased in the medium, cells take up more iron. Additionally, magnetite crystals in AMB-1 cells increase in size as iron concentration is increased. Previous genomic studies have shown that iron uptake genes are upregulated under magnetosome-forming conditions (18, 19, 21). However, it is not known if there is a genetic response to stimulate increased iron uptake or regulate the size of magnetite crystals in response to iron concentration. Here, we performed a magnetic selection with ML2 after growing cells at standard (30 μM) and high (150 μM) iron concentrations to uncover genes involved in magnetosome formation when cells are saturated with iron.

After magnetic selection, we compared the MCS from the magnetic populations of the standard and high-iron concentrations against each other (Fig. 3B). Most of the MCS either clustered around 0, meaning there was no measurable magnetic defect, or along the slope of 1, meaning there was no difference in magnetic response for that gene between the two iron conditions. There were several genes of interest that were underrepresented (had a magnetic defect) in the magnetic population of each condition. Under the standard iron condition, *amb0360* (AMB_RS01830), which is annotated as a hypothetical protein, had a magnetic defect. While there were no protein predictions, *amb0360* does have a homolog in Magnetospirillum gryphiswaldense MSR-1 (95.96% identity). One gene, *amb4208* (AMB_RS21290), was overrepresented (had a positive MCS) under the high-iron condition. It is possible that mutants of this gene are more magnetic than wild-type cells. Again, AMB4208 is a hypothetical protein with homologs in *Magnetospirillum* sp. strains XM-1 (81.5% identity), ME-1 (80.5% identity), and MSR-1 (75.54% identity).

There were several other genes with magnetic defects under the high-iron condition. Of note, there were several MAI genes that were underrepresented in the magnetic population: *mms6*, *mmsF*, *mamS*, and *mamT*. MamT is a magnetosome-localized protein with a magnetochrome domain (a heme-binding motif unique to magnetosome proteins) and is thought to be involved in redox chemistry for magnetosome crystal growth (26). MmsF and MamS are also magnetosome-localized proteins and have been shown to regulate crystal size and morphology (11, 12, 27). The lower magnetic score of these genes under the high-iron condition compared to the standard iron condition suggests that they have a more nuanced function in magnetosome formation than previously thought.

### Magnetic selection reveals genes important for biomineralization under anaerobic conditions.

Both AMB-1 and MSR-1 are capable of growing under microaerobic conditions. However, biomineralization only occurs when oxygen concentrations in the medium are depleted (23). In the laboratory, AMB-1 is typically grown with a low concentration of oxygen (2 to 10%) in the culture headspace, with nitrate as an alternative electron acceptor. It is not known if the inability of MTB to form magnetosomes aerobically is simply due to the balance of ferrous and ferric iron required to produce magnetosomes or if there is also a genetic response to turn off magnetosome production at high oxygen concentrations.

Here, we grew ML2 under either microaerobic (test tubes with minimal headspace incubated in a microaerobic chamber) or anaerobic (sealed serum bottles with anaerobic media) conditions and performed a magnetic selection to find genes that are required for magnetosome formation in various oxygen concentrations. The microaerobic conditions reflect how cells are grown under standard lab conditions.

We compared the MCS from the magnetic populations of the microaerobic and anaerobic samples against each other after magnetic selection (Fig. 3A). Again, most of the MCS either clustered around 0 or along the slope of 1, meaning there was no difference in magnetic response for most genes between the two oxygen conditions. Under the microaerobic condition, *amb0360* again had a magnetic defect, suggesting that it is important for biomineralization under standard conditions. Another gene, *amb4151* (AMB_RS21005), which is annotated as a hypothetical protein, had a positive MCS under the anaerobic condition, suggesting that mutants of this gene are more magnetic than wild-type cells. *amb4151* has homologs in *Magnetospirillum* sp. strain XM-1 (89.07% identity), ME-1 (87.89% identity), and MSR-1 (77.66% identity).

Several MAI genes also had magnetic defects under the anaerobic condition: *amb0936* (AMB_RS04810), *amb0947* (AMB_RS04850), *amb1008* (AMB_RS05170), *amb1009* (AMB_RS05175), *amb1022* (AMB_RS23960), *mamT*, *mmsF*, and *mamJ*. Again, it was surprising that *mamJ* came up in this screen, as MamJ is known for its role in chain organization, not biomineralization. While both *mamT* and *mmsF* deletion strains have defects in biomineralization (26, 27), it is surprising that the MCS is even lower under the high-iron condition because magnetosomes are typically larger in size under high-iron conditions. These results provide further support that the roles of some MAI genes are conditional.

### MamT plays a greater role for biomineralization under high-iron and anaerobic conditions.

**FIG 4:**
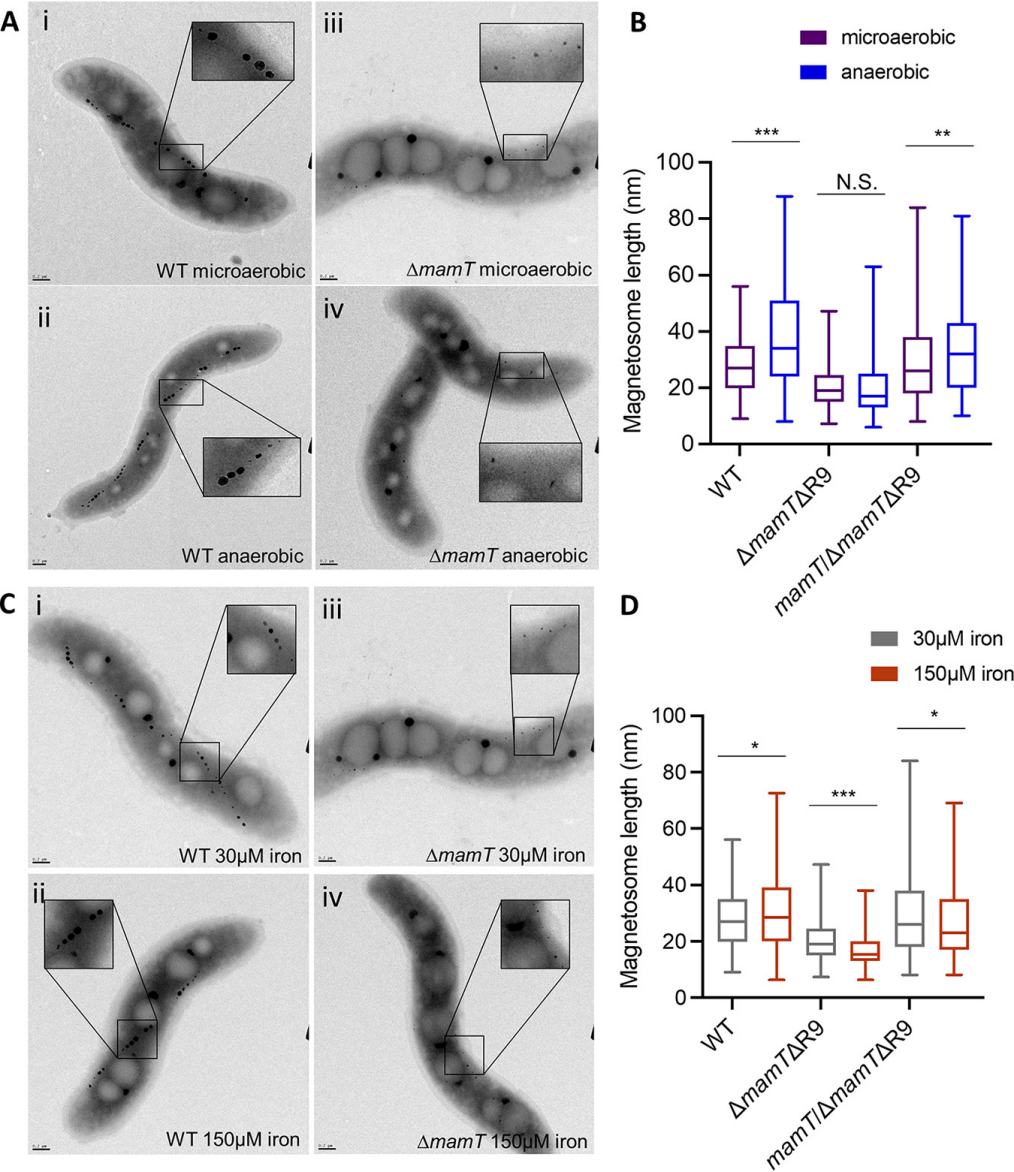
Validation of Δ*mamT* biomineralization phenotypes. (A) Representative images of WT and Δ*mamT*Δ*R9* cells under microaerobic (<10% oxygen) or anaerobic (0% oxygen) conditions. (B) Magnetosome length of WT (*n* = 292, *n* = 261), Δ*mamT*ΔR9 (*n* = 289, *n* = 204), and *mamT*/Δ*mamT*ΔR9 (*n* = 238, *n* = 230) magnetosomes after growth under microaerobic (<10% oxygen) or anaerobic (0% oxygen) conditions. (C) Representative images of WT and Δ*mamT*Δ*R9* cells under 30 μM iron or 150 μM iron conditions. (D) Magnetosome length of WT (*n* = 292, *n* = 284), Δ*mamT*ΔR9 (*n* = 289, *n* = 264), and *mamT*/Δ*mamT*ΔR9 (*n* = 238, *n* = 255) magnetosomes after growth in 30 μM iron or 150 μM iron.

To validate the results of the screen, we chose to look more closely at a *mamT* deletion to confirm that phenotypes seen in the magnetic selection screen also occur when the gene is deleted from a WT background. Here, we used the *ΔmamTΔR9* strain, where region 9 (R9) of the MAI (containing exact duplications of *mamQ*, *mamR*, and *mamB*) was also deleted to prevent a recombination event that makes the *mamT* deletion less stable (26). When grown with 30 μM iron, *ΔmamTΔR9* cells produce smaller crystals and have a very low magnetic response compared to WT cells (Fig. 4A and C). When grown with 150 μM iron, TEM images of *ΔmamTΔR9* cells qualitatively look very similar to cells grown with 30 μM iron (Fig. 4C). However, the length of the magnetite crystals is decreased in *ΔmamTΔR9* cells in 150 μM iron compared to 30 μM iron. As described above, it is surprising that magnetosomes would be smaller under the high-iron condition. Complementing the *ΔmamTΔR9* strain by integrating *mamT* into the genome restores the WT phenotype under both iron conditions. Again, we validated the phenotype of the *ΔmamTΔR9* strain under anaerobic conditions. When grown anaerobically, *ΔmamTΔR9* magnetite crystals were shorter than both wild-type and *ΔmamTΔR9* crystals under microaerobic conditions (Fig. 4A).

All experiments were repeated in the *ΔR9* strain to confirm that phenotypes seen with the *ΔmamTΔR9* strain were not caused by the R9 deletion (see Fig. S1 in the supplemental material). *ΔR9* cells behaved similarly to the WT in that they had larger magnetosomes under both high-iron and anaerobic conditions. The MAI gene *mms6* had a biomineralization defect under the 30 μM iron condition (−1.59 MCS), as expected (28), and slightly less of a defect under the 150 μM iron condition (−1.29 MCS), so we looked at *Δmms6* to verify that the magnetic selection was accurate and as a control for the *ΔmamTΔR9* strain. After growing the *Δmms6* strain in 30 μM or 150 μM iron, magnetosomes were larger under the high-iron conditions, similar to the WT (Fig. S2).

10.1128/mSystems.01037-21.1 FIG S1 Δ*R9* does not impact the Δ*mamT*Δ*R9* phenotype. (A) Magnetosome length of WT (*n* = 292, *n* = 261), Δ*mamT*Δ*R9* (*n* = 289, *n* = 204), and Δ*R9* (*n* = 204, *n* = 206) magnetosomes after growth in microaerobic (<10% oxygen) or anaerobic (0% oxygen) conditions. (B) Magnetosome length of WT (*n* = 292, *n* = 284), Δ*mamT*Δ*R9* (*n* = 289, *n* = 264), and Δ*R9* (*n* = 204, *n* = 235) magnetosomes after growth under 30 μM iron or 150 μM iron conditions. Download FIG S1, TIF file, 2.6 MB. Copyright © 2022 McCausland et al. 2022 McCausland et al. https://creativecommons.org/licenses/by/4.0/ This content is distributed under the terms of the Creative Commons Attribution 4.0 International license.

10.1128/mSystems.01037-21.2 FIG S2 Δ*mms6* magnetosomes are longer under high-iron conditions. (A) Representative transmission electron micrographs of WT and Δ*mms6* strains grown in 30 μM or 150 μM iron. (B) Magnetosome length of WT (*n* = 292, *n* = 284), Δ*mamT*Δ*R9* (*n* = 289, *n* = 264), and Δ*mms6* (*n* = 235, *n* = 238) magnetosomes after growth under 30 μM iron or 150 μM iron conditions. Download FIG S2, TIF file, 2.6 MB. Copyright © 2022 McCausland et al. 2022 McCausland et al. https://creativecommons.org/licenses/by/4.0/ This content is distributed under the terms of the Creative Commons Attribution 4.0 International license.

These results together suggest that the magnetic selection process is sufficient for pulling out genes that, when disrupted, have a defect in biomineralization. They also show that *mamT*, which was already known to be involved in magnetosome formation, may have a more critical role under both high-iron and anaerobic conditions.

### Genes outside the MAI may have a role in biomineralization.

**FIG 5:**
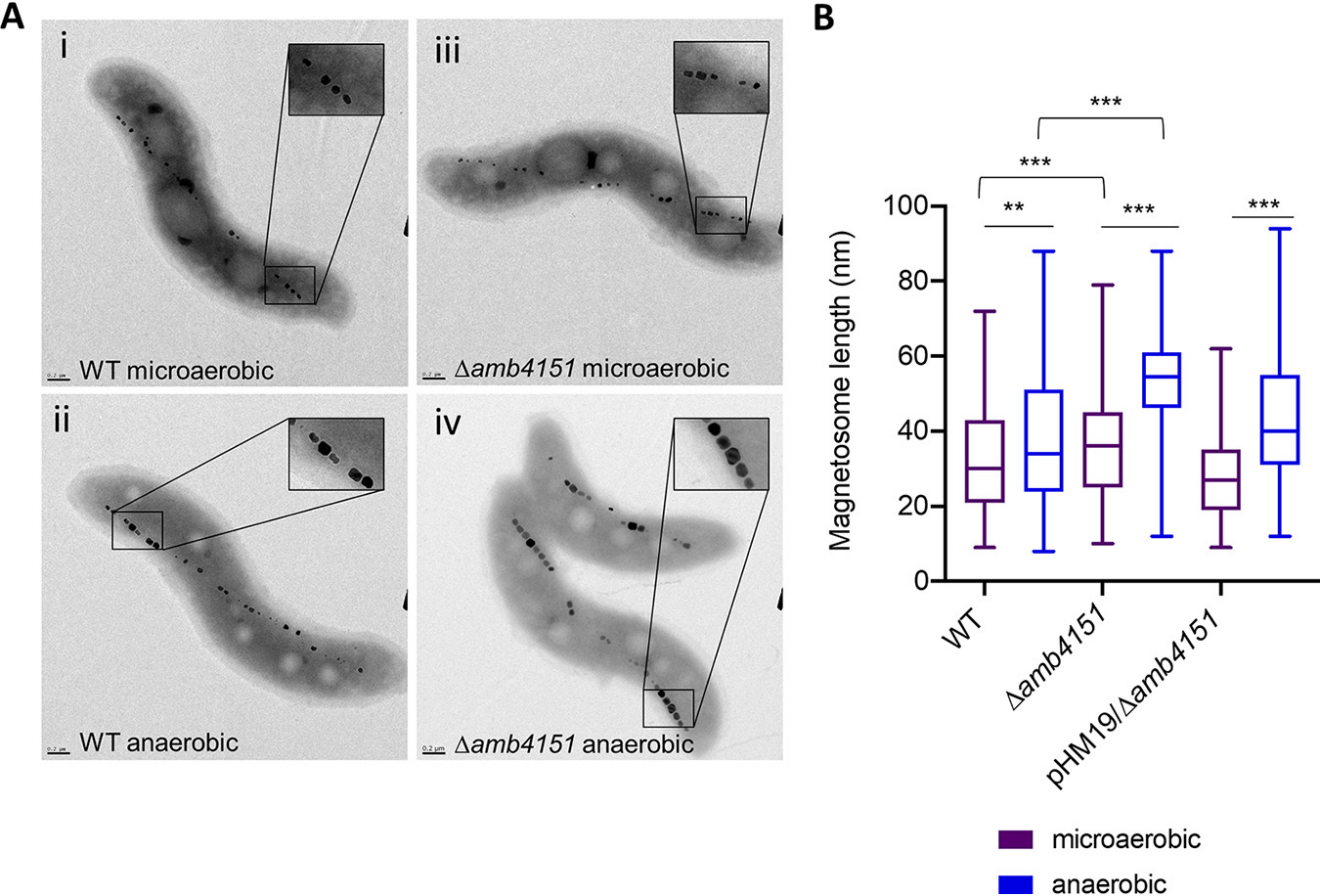
Validation of Δ*amb4151* biomineralization phenotypes. (A) Representative transmission electron micrographs of WT and Δ*amb4151* strains grown under microaerobic (<10% oxygen) and anaerobic (0% oxygen) conditions. (i) WT grown under microaerobic conditions. (ii) WT grown under anaerobic conditions. (iii) Δ*amb4151* strain grown under microaerobic conditions. (iv) Δ*amb4151* strain grown under anaerobic conditions. (B) Magnetosome length of WT (*n* = 234, *n* = 230), Δ*amb4151* (*n* = 261, *n* = 250), and *amb4151*/Δ*amb4151* (*n* = 222, *n* = 202) magnetosomes after growth under microaerobic or anaerobic conditions.

It was unexpected to see a gene with a positive MCS under the anaerobic condition, especially a gene outside the MAI, as this indicates that a novel ex-MAI gene has a role in control of biomineralization. To explore this phenomenon further, we generated a deletion of *amb4151*. Under microaerobic conditions, *Δamb4151* magnetosomes looked similar to WT magnetosomes (Fig. 5A) and had the same magnetosome length (Fig. 5B). Under anaerobic conditions, WT AMB-1 cells typically produce larger magnetosomes (mean, 37.2 nm; σ = 17.0). *Δamb4151* cells produced larger magnetosomes than the WT under anaerobic conditions and appeared to do so more consistently (mean, 53.5 nm; σ = 15.8).

## DISCUSSION

Previous genetic studies in MTB have relied on transposon mutagenesis screens that required sorting through thousands of individual colonies (29–31). Here, we show that RB-TnSeq is a streamlined and effective technique for investigating biomineralization in MTB, as the pooled library allows for screening hundreds of thousands of mutants through many growth conditions in a relatively short amount of time. Additionally, the method is sensitive to small changes in magnetic properties of a mutant. These features allowed us to demonstrate that some known magnetosome genes have differential roles as growth conditions change and that novel genes outside the MAI may have an impact on biomineralization.

There are clear benefits to using RB-TnSeq over traditional methods of mutagenesis. First, the simplicity of sequencing barcodes in each sample allows for streamlined screening and the ability to test many different conditions in a single experiment. Additionally, because the phenotypic score for a gene is averaged across many individual insertions, we can be more confident that genes of interest are worth pursuing. However, RB-TnSeq is not without its challenges. Because all mutants are pooled, it is possible that some phenotypes of some mutants may be masked via transcomplementation by other mutants in the pool or by the availability of common goods (i.e., siderophores and signaling molecules). Alternatively, it may be difficult to validate the phenotype after screening. There is a chance that a mutant is influenced by competition in the pool, for example, the mutants of one gene are outcompeted for nutrient uptake and form smaller magnetosomes, and once the gene is examined on its own, the phenotype may disappear. Biased insertion and polar effects could also cause false positives in some instances. A phenotype can still be evaluated after the fact with single gene or operon deletions. As an example of this phenomenon, polar effects may explain why insertions in *mamJ* caused magnetic defects, even though previous results have indicated that *mamJ* deletion does not have an effect on biomineralization (32, 33).

In this study, the ML2 (wild-type) library had full coverage of the genome, with over 180,000 strains and approximately 34 transposon insertions per gene. With ML2 we were able to map the essential gene set of AMB-1 under our standard laboratory conditions, which are the conditions under which most genetic studies have been conducted in AMB-1. The essential gene set expands our understanding of the physiology of AMB-1 and allows for comparison to other MTB in either physiological or phylogenetic studies.

We then screened the transposon mutant pool using a magnetic selection. Previous studies in MTB have used similar selection methods (i.e., colony color, response to a magnetic field, and magnetic column) (29–31, 34, 35), but the barcode sequencing adds power by quantifying the results. Since MCS is based on a weighted average of all the mutants in a particular gene, a biomineralization phenotype for a particular gene is more reliable. As the biomineralization phenotype for one strain can vary across the population of cells, averaging the scores of multiple mutant strains provides more confidence that the phenotype is real. Additionally, ML2 being a complete library allows us to reexamine mutants that were previously discovered with standard techniques.

Mutants with insertions in MAI genes were depleted from the population of magnetic cells after magnetic selection, indicating that the screening method reliably detects strains with defects in biomineralization. Interestingly, genes identified in a previous transposon mutagenesis screen by Matsunaga et al. (17) as being essential for biomineralization were not found to have biomineralization defects in this study. Matsunaga et al. created 5,762 Tn*5* transposon mutants and found 69 mutants with defects in biomineralization, none of which were located in the MAI. It is possible there were secondary mutations in the Tn*5* transposon mutants that were the true cause of the biomineralization defect, for example, spontaneous loss of the MAI. One benefit of RB-TnSeq is that the phenotype is based on an average of multiple insertions in each gene, making it less likely to falsely link a gene to biomineralization.

We also discovered potential new functions for known magnetosome genes when cells were grown under nonstandard conditions (high iron or anaerobic). Of note was *mamT*, which is known to be involved in crystal formation but may be more critical under high-iron or anaerobic conditions. The *mamT* phenotype is notable because under high-iron or anaerobic conditions we expect magnetosomes to get larger, and *mamT* mutants have the opposite phenotype. The effect on magnetosome size was also quite subtle, which highlights the power of the magnetic screen and its ability to identify phenotypes that would have been overlooked otherwise. While we did not investigate the mechanism in this work, perhaps the decreased size of magnetosomes under those two conditions are due to the proposed role of MamT in regulating the balance of iron species within the magnetosome. Further study of MamT under alternative growth conditions could provide further insight into its function.

While some of the genes in the MAI are transposable elements and are unlikely to have a role in magnetosome formation, the screens here showed that many of the genes in the MAI originally thought to have no role in biomineralization, or that have not been studied before, may be needed as conditions shift. These genes include *amb0936*, *amb0947*, *amb0958*, *amb0959*, *amb1009*, *amb1008*, *amb1022*, *mamJ*, *mamK*, and *mamY*. It is especially surprising that *mamJ* mutants had a biomineralization defect, since the phenotype of *mamJ* is masked by the presence of *limJ* in the magnetosome islet (36). Additionally, we uncovered genes outside the MAI that may have an impact on magnetosome formation. We found that *amb4151*, annotated as a hypothetical protein, inhibits biomineralization to a small degree. The *Δamb4151* strain has larger magnetosomes under anaerobic conditions than the WT.

It has been shown that magnetosomes are only made under low oxygen concentrations (23). However, it is not known if this is simply due to shifting redox conditions or if there is also a genetic response to turn off magnetosome production at high oxygen concentrations. Further study of *amb4151* could be useful to understand the impact of ex-MAI genes on magnetosome formation. The phenotype of *amb4151* also suggests that the changes in magnetosome size under shifting iron and oxygen increases not only are due to redox conditions but also are influenced by a genetic response.

The genes highlighted in this study all had nuanced phenotypes, in that each gene deletion had a slight effect on biomineralization. While these genes may not be critical to the formation of magnetosomes, it is still valuable to study their functions. Most, if not all, of the genes that have a large impact on biomineralization or magnetosome formation have already been identified in past screens and reverse genetic studies. Genes with more subtle phenotypes can provide key information on the formation of magnetosomes under different conditions.

While previous studies have suggested that MTB have a large environmental impact, we know very little about the dynamics of magnetosome formation in natural environments. A greater understanding of the molecular mechanisms of biomineralization can help with understanding how much iron is taken up by MTB and under what conditions. This study shows that RB-TnSeq and screens for biomineralization defects are useful tools to identify genes that influence the response of AMB-1 to changing environmental conditions. There are certainly many applications for this technique to reveal more about growth and biomineralization in MTB. For example, an RB-TnSeq library may be used to find genes that are necessary in an oxygen or redox gradient. Taken a step further, it could be used to find mutants in magnetoaerotaxis. Both screens would reveal more about the physiology and lifestyle of magnetotactic bacteria.

## MATERIALS AND METHODS

### Growth and culture conditions.

*Magnetospirillum magneticum* AMB-1 was cultured in defined minimal medium (MG medium) supplemented with a 1/100 volume of Wolfe’s vitamin solution and 30 μM ferric malate as previously described (31). Colonies were grown on solid MG with 0.7% agar. Kanamycin was used at 10 μg/ml in solid medium and 7 μg/ml in liquid medium for strains with a kanamycin-resistant cassette integrated into the chromosome or on a plasmid (Table 1). For microaerobic growth, cells were grown in culture tubes or 50-ml conical tubes and incubated at 30°C in a microaerobic chamber with 10% oxygen. For anaerobic growth, sealed Balch tubes containing 10 ml MG medium and 20 ml headspace were used. Medium was bubbled with N_2_ gas for 10 min before sealing. The headspace then was flushed with N_2_ gas for 10 min before autoclaving. Ferric malate and Wolfe’s vitamins were added once tubes cooled. All cultures were inoculated with a dilution factor of 1:100.

**TABLE 1:** Plasmids

Name	Origin	Description	Source
pHM14	pAK31-derived	Deletion plasmid for *amb4151*	This work
pHM19	pAK22-derived	Complementation plasmid for Δ*amb4151*	This work
pAK807	pAK605-derived	Complementation plasmid for Δ*mamT*Δ*R9*	Patrick Browne (unpublished)
pHM20	pAK31-derived	Deletion plasmid for *amb3952*	This work

### Library construction and processing.

Detailed description of transposon vector construction can be found in Wetmore et al. (22). AMB-1 mutant libraries were constructed using APA752 (*mariner* transposon library pKMW3 in WM3064 containing millions of unique 20-nucleotide barcodes). APA752 was transferred to AMB-1 from WM3064 by conjugation: 1 liter of wild-type AMB-1 culture at an OD_400_ of 0.230 was conjugated with 100 ml of APA752 culture by incubating in a microaerobic (10% oxygen) chamber for 14 h, diluting 1:15, and plating on MG/Kan. Plates were incubated in microaerobic jars (7% oxygen) at 30°C for 5 days. Colonies were collected from plates, pooled in liquid MG/Kan, and then allowed to grow to late log phase before spinning down 50-ml aliquots and freezing in 1-ml aliquots with 20% glycerol. All libraries were made in standard MG. Some cell pellets from the 50-ml aliquots were set aside for gDNA extraction. To ensure that the library had enough barcodes to indicate complete coverage of the genome, BarSeq PCR followed by Illumina MiSeq sequencing was performed. Libraries determined to be diverse enough were sequenced by Illumina HiSeq to map transposon insertions. Illumina library preparation and TnSeq data analysis are described in Wetmore et al. (22).

### Essential gene analysis.

We used previously published methods to determine the essential gene set of AMB-1 in both standard wild-type and *ΔMAI* strains using the RB-TnSeq libraries (25). Briefly, read density was determined for each protein-coding gene. Genes that are very similar to other parts of the genome and genes of less than 100 nucleotides were excluded from analysis. Based on the median insertion density and median length of remaining genes, a threshold for gene size included in the essential gene analysis was set for the library by determining how short a gene could be and still be unlikely to have no insertions at all by chance (*P < *0.02, Poisson distribution) (25). The threshold for ML2 was 800 nucleotides; any genes shorter than that were excluded from the essential gene analysis.

### Competitive mutant magnetic assays.

For each condition, one aliquot of ML2 was thawed and inoculated into 250 ml of MG with 7 μg/ml kanamycin. When the culture recovered to stationary phase at an OD_400_ of ∼0.250 on an Ultrospec 2100 pro (Amersham), cell pellets were collected from 25-ml aliquots as time zero samples. For experiments testing iron concentrations, cells were first washed twice and resuspended in MG without carbon or iron. Cultures then were inoculated at 1:100 in triplicate for each condition. For iron experiments, MG was supplemented to either 30 μM or 150 μM ferric iron using 3 mM ferric malate stock. Cultures were balanced with malic acid (18 mM [200×] stock). For oxygen experiments with ML2, microaerobic cultures were grown in culture tubes in a microaerobic glove box without shaking, and anaerobic cultures were grown in Balch tubes without shaking.

### Magnetic selection.

Samples of 15 ml of culture from each condition and each triplicate were pelleted as the precolumn sample and frozen at −20°C. For each magnetic selection, an LS column (Miltenyi Biotech) was set up on a ring stand with 6 sets of 2 neodymium N52 magnets on each side. The remaining 35 ml of culture was passaged over the magnetic column via gravity filtration and collected in a 50-ml conical tube (magnetic sample). The magnets then were removed and the column was washed twice with 5 ml of MG. Flowthrough (nonmagnetic sample) was collected. Both magnetic and nonmagnetic samples were spun down, and cell pellets were frozen at −20°C.

### BarSeq.

Genomic DNA was isolated from library samples using the Qiagen DNeasy blood and tissue kit. gDNA concentration was quantified by NanoDrop. BarSeq PCR was performed using ∼200 ng of DNA for each sample. Barcodes were amplified using unique, indexed primers, as described in Liu et al. (37). Samples were pooled and sequenced using Illumina HiSeq single-end reads.

### BarSeq data analysis and magnetic column abundance calculation.

A detailed description of BarSeq analysis can be found in Wetmore et al. (22). Briefly, the MCS for a gene is calculated from the weighted average of the strain MCS. BarSeq data can be viewed in the Fitness Browser (http://fit.genomics.lbl.gov), which provides information from successful selection experiments, including details of the experimental conditions, quality metrics for each experiment, per-strain scores, and gene scores (25). Because these methods were developed for use with growth assays, we added an additional normalization step to account for any growth defects that might influence MCS after passing cells through the magnetic column by subtracting precolumn gene MCS from magnetic and nonmagnetic gene MCS.

### Strains.

*ΔmamTΔR9* (26), Δ*mmsF* (27), and Δ*mms6* (27) strains were generated previously (Tables 2 and 3). All other gene deletions were made using a two-step recombination method to generate a markerless deletion (31). Deletion plasmids were constructed using three-piece Gibson assembly. Regions upstream and downstream of the gene of interest were cloned into pAK31 (suicide vector containing the kanamycin resistance cassette and *sacB* gene) cut with BamHI and SpeI. The plasmids were transferred to AMB-1 cells by conjugation and selected on MG/kanamycin medium. Colonies were then grown on MG with 2% sucrose to select for deletion strains. Sucrose-resistant colonies were screened by PCR for the deletion and for the lack of plasmid markers.

**TABLE 2:** Strains

Strain	Organism	Description	Reference or source
APA752	E. coli	Mariner RB-TnSeq library	22
AK155	AMB-1	*ΔmamTΔR9*	26
AK57	AMB-1	*ΔR9*	11
AK104	AMB-1	Δ*mmsF*	27
AK110	AMB-1	Δ*mms6*	27
HM02	AMB-1	Δ*amb4151*	This work
DH5α (λpir)	E. coli	Standard molecular cloning strain	
WM3064	E. coli	Plasmid conjugation strain	W. Metcalf, University of Illinois, Urbana

**TABLE 3:** Primers

Name	Sequence	Use
HM138	CGAATTCCTGCAGCCCGGGGAAATTCAGAAAATCATAAAATCCACC	Δ*amb4151*
HM139	AAGTCCAGTTCTCTTGCAAATCCGTCGG	Δ*amb4151*
HM140	TTTGCAAGAGAACTGGACTTTACCCTTGCGG	Δ*amb4151*
HM141	CGGTGGCGGCCGCTCTAGAAGACGCCCTTGGCGGTCAG	Δ*amb4151*
HM146	CAATTTCACACAGGAAACAGATGATGATCAGTCACCACGCGGC	pHM19
HM147	CGGTGGCGGCCGCTCTAGAATCAGGCGTGCACCGGCAG	pHM19

Complementation plasmids were constructed using three-piece Gibson assembly. The gene of interest was cloned into pAK22 (replicative plasmid containing the kanamycin resistance cassette following a *tac* promoter) cut with EcoRI and SpeI. The plasmids were transferred to AMB-1 cells by conjugation and selected on MG/kanamycin medium.

### TEM.

Whole AMB-1 cells were imaged by transmission electron microscopy (TEM). To prepare samples for imaging, 1.5 ml of stationary-phase (OD_400_ of ∼0.25) culture was pelleted and resuspended in 10 μl of MG. Cells were absorbed on glow-discharged, 200-mesh Cu grids with Formvar film and imaged on an FEI Technai 12 transmission electron microscope with a Gatan Bioscan (1,000 by 1,000) charge-coupled device (CCD) camera model 792 at an accelerating voltage of 120 kV.

### Quantification of crystal size and magnetosome numbers.

TEM images were used to measure crystal size and numbers. The length and width of each crystal was measured along its long axis by hand using ImageJ. More than 200 magnetosomes were measured for each strain and condition.

10.1128/mSystems.01037-21.3 TABLE S1 Test for normality in magnetosome length analysis. All datasets were analyzed for normality using the Shapiro-Wilk test. If the *P* value is smaller than 0.05, the data tested do not form a normally distributed population. Download Table S1, DOCX file, 0.01 MB. Copyright © 2022 McCausland et al. 2022 McCausland et al. https://creativecommons.org/licenses/by/4.0/ This content is distributed under the terms of the Creative Commons Attribution 4.0 International license.

10.1128/mSystems.01037-21.4 TABLE S2 Significance difference tests between magnetosome length datasets. If the datasets are normally distributed, unpaired Student *t* tests were performed; if one or both datasets are randomly distributed, Mann-Whitney U test was used. For either test, if the *P* value is less than 0.05, the difference between the two analyzed samples is considered statistically significant. Download Table S2, DOCX file, 0.02 MB. Copyright © 2022 McCausland et al. 2022 McCausland et al. https://creativecommons.org/licenses/by/4.0/ This content is distributed under the terms of the Creative Commons Attribution 4.0 International license.

10.1128/mSystems.01037-21.5 DATA SET S1 Magnetic column scores (MCS) for all conditions and replicates discussed in this study. AMB-1 genes are listed by RefSeq ID and annotation. Column C includes time 0 scores. Replicates for each condition and sample (precolumn, magnetic, and nonmagnetic) are listed in a separate column, followed by the mean of replicate samples. Cells highlighted in blue indicate a negative MCS, and cells highlighted in green indicate a positive MCS. Genes highlighted in orange are part of the magnetosome gene island (MAI). Download Data Set S1, XLSX file, 0.4 MB. Copyright © 2022 McCausland et al. 2022 McCausland et al. https://creativecommons.org/licenses/by/4.0/ This content is distributed under the terms of the Creative Commons Attribution 4.0 International license.
