# Supplementary figures and images for: Global Analysis of Biomineralization Genes in Magnetospirillum magneticum AMB-1
Source: mSystems. 2022 Jan 25;7(1):e01037-21. doi: 10.1128/msystems.01037-21 (PMC8788322; doi:10.1128/msystems.01037-21)

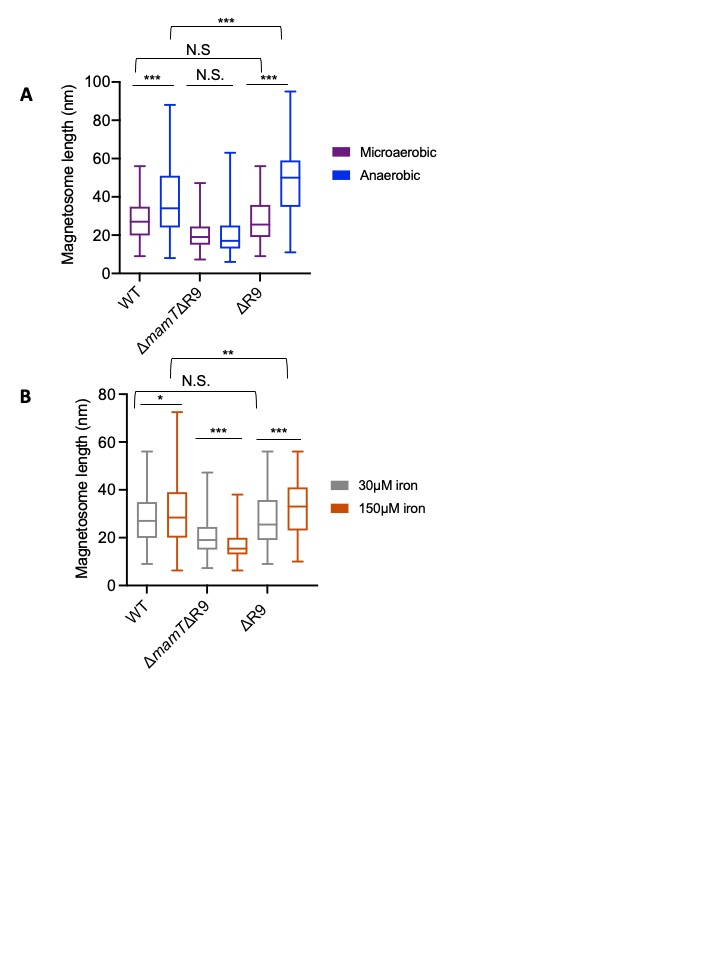

Supplement: FIG S1 [file msystems.01037-21-sf001.tif]

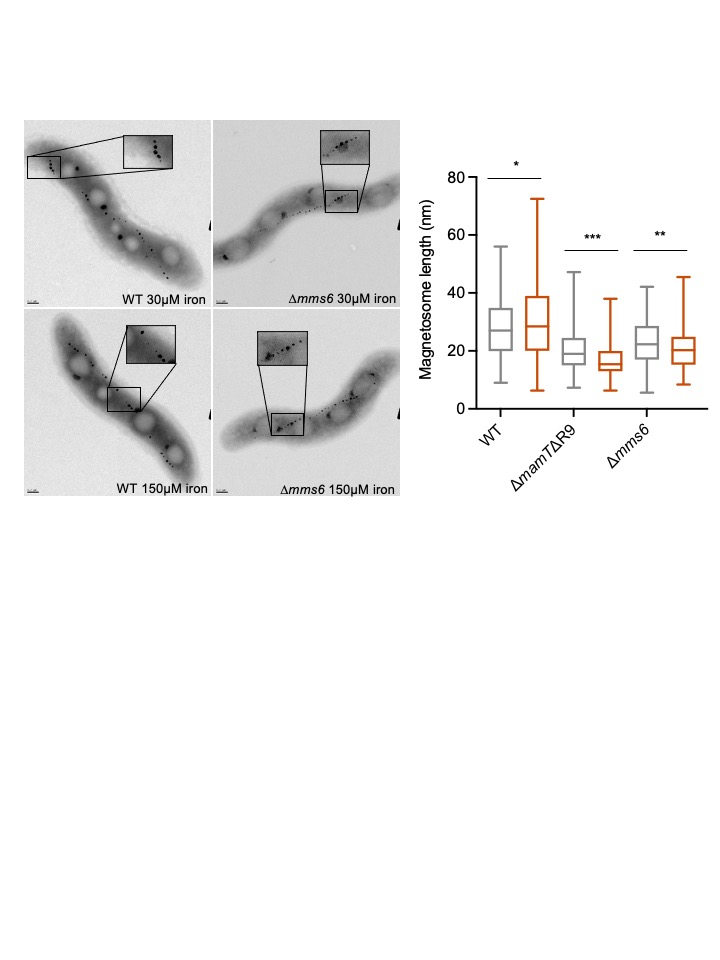

Supplement: FIG S2 [file msystems.01037-21-sf002.tif]
