# Supplementary material for: Global Analysis of Biomineralization Genes in Magnetospirillum magneticum AMB-1
Source: mSystems. 2022 Jan 25;7(1):e01037-21. doi: 10.1128/msystems.01037-21 (PMC8788322; doi:10.1128/msystems.01037-21)
Supplement: TABLE S1 [file msystems.01037-21-st001.docx]

**Table S1**—Test for normality in magnetosome length analysis

| Figure | Strain, Condition | Shapiro-Wilk normality test (P-value) |
| --- | --- | --- |
| Fig. 4B | WT, microaerobic | 0.0054 |
|  | WT, anaerobic | <0.0001 |
|  | *∆mamT∆R9*, microaerobic | <0.0001 |
|  | *∆mamT∆R9*, anaerobic | <0.0001 |
|  | *mamT*/*∆mamT∆R9,* microaerobic | <0.0001 |
|  | *mamT*/*∆mamT∆R9,* anaerobic | <0.0001 |
| Fig. 4D | WT, 30 μM | 0.0054 |
|  | WT, 150 μM | <0.0001 |
|  | *∆mamT∆R9*, 30 μM | <0.0001 |
|  | *∆mamT∆R9*, 150 μM | <0.0001 |
|  | *mamT*/*∆mamT∆R9*, 30 μM | <0.0001 |
|  | *mamT*/*∆mamT∆R9*, 150 μM | <0.0001 |
| Fig. 5B | WT, microaerobic | <0.0001 |
|  | WT, anaerobic | <0.0001 |
|  | *∆amb4151*, microaerobic | 0.002 |
|  | *∆amb4151*, anaerobic | <0.0001 |
|  | pHM19/*∆amb4151,* microaerobic | <0.0001 |
|  | pHM19/*∆amb4151,* anaerobic | <0.0001 |
| Fig. S1A | WT, microaerobic | 0.0054 |
|  | WT, anaerobic | <0.0001 |
|  | *∆mamT∆R9*, microaerobic | <0.0001 |
|  | *∆mamT∆R9*, anaerobic | <0.0001 |
|  | *∆R9,* microaerobic | <0.0001 |
|  | *∆R9,* anaerobic | 0.0238 |
| Fig. S1B | WT, 30 μM | 0.0054 |
|  | WT, 150 μM | <0.0001 |
|  | *∆mamT∆R9*, 30 μM | <0.0001 |
|  | *∆mamT∆R9*, 150 μM | <0.0001 |
|  | *∆R9*, 30 μM | <0.0001 |
|  | *∆R9*, 150 μM | 0.0003 |
