# Supplementary material for: Global Analysis of Biomineralization Genes in Magnetospirillum magneticum AMB-1
Source: mSystems. 2022 Jan 25;7(1):e01037-21. doi: 10.1128/msystems.01037-21 (PMC8788322; doi:10.1128/msystems.01037-21)
Supplement: TABLE S2 [file msystems.01037-21-st002.docx]

**Table S2**—Significance difference tests between magnetosome length datasets

|  | | **Mann-Whitney U test** | | **t-test** | |
| --- | --- | --- | --- | --- | --- |
| **Figure** | **Strains/Conditions** | **p-value** | **Significant difference** | **p-value** | **Significant difference** |
| Fig. 4B | WT microaerobic & WT anaerobic | 0.030 | * | ---- | ---- |
|  | *∆mamT∆R9* microaerobic & *∆mamT∆R9* anaerobic | 0.061 | * | ---- | ---- |
|  | *mamT*/*∆mamT∆R9* microaerobic & *mamT*/*∆mamT∆R9* anaerobic | 0.007 | ** | ---- | ---- |
|  | WT microaerobic & *∆mamT∆R9* microaerobic | <0.0001 | **** | ---- | ---- |
|  | WT anaerobic & *∆mamT∆R9* anaerobic | <0.0001 | **** | ---- | ---- |
|  | WT microaerobic & *mamT/∆mamT∆R9* microaerobic | 0.580 | N.S. | ---- | ---- |
|  | WT anaerobic & *mamT/∆mamT∆R9* anaerobic | 0.023 | * | ---- | ---- |
| Fig. 4D | WT 30 μM & WT 150 μM | 0.030 | * | ---- | ---- |
|  | *∆mamT∆R9* 30 μM & *∆mamT∆R9* 150 μM | <0.0001 | **** | ---- | ---- |
|  | *mamT*/*∆mamT∆R9* 30 μM & *mamT*/*∆mamT∆R9* 150 μM | 0.041 | * | ---- | ---- |
|  | WT 30 μM & *∆mamT∆R9* 30 μM | <0.0001 | **** | ---- | ---- |
|  | WT 150 μM & *∆mamT∆R9* 150 μM | <0.0001 | **** | ---- | ---- |
|  | WT 30 μM & *mamT/∆mamT∆R9* 30 μM | 0.580 | N.S. | ---- | ---- |
|  | WT 150 μM & *mamT/∆mamT∆R9* 150 μM | 0.0004 | *** | ---- | ---- |
| Fig. 5B | WT microaerobic & WT anaerobic | 0.0015 | ** | ---- | ---- |
|  | *∆amb4151* microaerobic & *∆amb4151* anaerobic | <0.0001 | **** | ---- | ---- |
|  | pHM19/*∆amb4151* microaerobic & pHM19/*∆amb4151* anaerobic | <0.0001 | **** | ---- | ---- |
|  | WT microaerobic & *∆amb4151* microaerobic | 0.007 | ** | ---- | ---- |
|  | WT anaerobic & *∆amb4151* anaerobic | <0.0001 | **** | ---- | ---- |
|  | WT microaerobic & pHM19*/∆amb4151* microaerobic | 0.003 | ** | ---- | ---- |
|  | WT anaerobic & pHM19*/∆amb4151* anaerobic | <0.0001 | **** | ---- | ---- |
| Fig. S1A | *∆R9* microaerobic & *∆R9* anaerobic | <0.0001 | **** | ---- | ---- |
|  | WT microaerobic & *∆*R9 microaerobic | 0.828 | N.S. | ---- | ---- |
|  | WT anaerobic & *∆*R9 anaerobic | <0.0001 | **** | ---- | ---- |
| Fig. S1B | *∆R9* 30 μM & *∆R9* 150 μM | <0.0001 | **** | ---- | ---- |
|  | WT 30 μM & *∆*R9 30 μM | 0.828 | N.S. | ---- | ---- |
|  | WT 150 μM & *∆*R9 150 μM | 0.008 | ** | ---- | ---- |

Significantly different (*P<0.05, **P<10^-2^, ***P<10^-3^, ****P<10^-4^); N.S. not significantly different.
